# Psychometric properties of the Slovenian version of the Cancer Survivors’ Unmet Needs (CaSUN-SL) measure in post-treatment cancer survivors

**DOI:** 10.1186/s40359-022-00878-6

**Published:** 2022-07-17

**Authors:** Špela Miroševič, Polona Selič-Zupančič, Judith Prins, Vesna Homar, Zalika Klemenc-Ketiš

**Affiliations:** 1 Department of Family Medicine, Faculty of Medicine, University of Ljubljana, Poljanski nasip 58, 1000 Ljubljana, Slovenia; 2 Department of Psychology, Faculty of Medicine, University of Maribor, Maribor, Slovenia; 3 Primary Healthcare Research and Development Institute, Community Health Centre Ljubljana, Ljubljana, Slovenia; 4 Department of Medical Psychology, Radboud University Medical Centre, Nijmegen, The Netherlands

**Keywords:** Cancer survivors, Needs assessment, Psychometrics, Anxiety, Depression, Quality of life

## Abstract

**Background:**

As the number of cancer survivors is growing, valid instruments for assessing cancer survivors' needs are required. Thus, the aim of this study was to translate and validate the Cancer Survivors Unmet Needs (CaSUN) scale.

**Methods:**

Cancer survivors were recruited from 30 family medicine practices and separated into two samples (sample 1, n = 147; sample 2, n = 148). Factor structure was explored with an exploratory analysis in sample 1 and determined with a confirmatory analysis in sample 2. Psychometric properties were assessed with internal consistency, test–retest reliability and construct validity.

**Results:**

A translation and cultural adaptation of the CaSUN scale resulted in 34 items being included in the final version. The factor structure confirmed the five-factors solution of the CaSUN-SL. Cronbach’s alpha was 0.94 for the CaSUN-SL and ranged from 0.71–0.88 for specific domains. Test–retest reliability showed moderate-high stability over time. The CaSUN-SL significantly and positively correlated with anxiety (r = 0.49), depression (r = 0.44), health-related quality of life (r = 0.36), and negatively with self-perceived health (r =  − 0.36) and resilience (r =  − 0.47), which confirms the construct validity. In addition, we found a significant correlation between unmet needs and age (r =  − 0.29), gender (r = 0.14), cancer stage (r = 0.20), cancer type (r = 0.19), and time since treatment (r =  − 0.20).

**Conclusions:**

Results indicate that CaSUN-SL is a valid and reliable measure to assess the Slovenian cancer survivors’ unmet, met and total needs and can be used for further prospective studies.

*Trial Registration:* No. 0120-25/2019/6.

## Background

Cancer and cancer treatment are associated with various physical and psychosocial problems, including difficulties with fatigue, declines in physical functioning, fear of cancer recurrence, anxiety, depression, cognitive limitations, coping issues, treatment-induced menopausal problems, and fertility problems, [1–4]. Although the majority of cancer survivors recover well, a significant number of them continue to have needs related to their disease and treatment [5, 6]. Cancer survivors who report having more unmet information and support needs also have more symptoms of depression and anxiety and a lower quality of life [7, 8]. It is important to address cancer survivors’ needs as longitudinal studies report that unaddressed needs affect patients’ quality of life [9], increase the frequency of patients’ visits to healthcare facilities [10], and most importantly, do not resolve by themselves but cause even more unmet needs in the future [11].

Several different instruments are available to assess cancer survivors’ unmet needs, including the Cancer Survivors’ Unmet Needs (CaSUN) scale [12], the Survivor Unmet Needs Survey (SUNS) [13] and its short form (SUNS-SF) [14], the Cancer Needs Questionnaire-Young People (CNQ-YP), the Needs Assessment Questionnaire (CCSS-NAQ) [15], and the 34-item Supportive Care Needs Survey (SCNS-34) [16] and its short form (SCNS-SF-34) [17]. Only the SUNS and the CaSUN are adult-survivors-specific measures. Reviews comparing different unmet needs measures reported that the SUNS has the most robust psychometric properties [18]; however, it can be less practical than the CaSUN due to its length (89 items) and a large number of items relating to financial issues. In countries (e.g., Slovenia) with a mandatory insurance scheme, covering concerns about quality-related unmet needs is more relevant than focusing on financial issues. The CaSUN was found to be the most comprehensive measure [18], as it covers the broadest area of unmet needs: emotional, spiritual, social, information, physical and practical [7].

The CaSUN was developed for the Australian cancer survivor’s population, where a good internal reliability was reported (Cronbach α: 0.78–0.93). The construct validity of the CaSUN was evaluated with the Hospital Anxiety and Depression Scale (HADS) and the 12-item Short-Form Health Survey (SF-12) for measuring quality of life, and it was found to have a positive association with both [12]. The majority of validation studies found a significant association between age and the total number of unmet needs [19–21]. Marital status, and cancer-related variables were found not to be associated with the total number of unmet needs [12, 20]. In 2016, the first translations of the CaSUN were completed in Dutch and Chinese [20, 21]. To date, the CaSUN was translated into Spanish [22], Japanese [19], and Korean [23] and has been used for cancer survivors living between 6 months and up to 15 years after the end of the treatment [24–26] with a diagnosis of breast [21, 27–29], colorectal [26, 30, 31], testicular [25], prostate [32], head and neck [33], non-small cell lung [23], gynaecologic [34, 35], endometrial [36], and a cohort of mixed cancer types [12, 19, 20, 24, 37–39].

In Slovenia, the area of cancer survivors’ needs is poorly described. There is an urgent need for linguistic translation of the already developed unmet need tools. Thus, the aim of this study is to first translate the English version of the CaSUN into the Slovenian language and second, to determine the psychometric properties (factor structure, internal consistency and test–retest reliability) of the CaSUN-SL. Construct validity is planned to be assessed with the Hospital Anxiety and Depression Scale (HADS) and the EuroQol Five-Dimension questionnaire (EQ-5D). Our hypothesis is that unmet needs would correlate positively with the symptoms of anxiety and depression, and health-related quality of life, and negatively with resilience and age [20, 25, 36]. In addition, we plan to explore the association between unmet needs and marital status, type of cancer and time since treatment in order to guide further research in the Slovenian population of cancer survivors [35, 36, 40].

## Methods

This study received approval from the National Medical Ethics Committee (no. 0120–25/2019/6) and was in compliance with the Declaration of Helsinki for recommendations guiding physicians in biomedical research involving human subjects. Informed consent was obtained from all the participants prior to their participation.

This cross-sectional study was conducted in three steps. First, we back-translated the CaSUN according to the recommendations [41]. In the second phase, we culturally adapted the scale and pilot tested it for content validity. Thus, the initial version of the Slovenian CaSUN (CaSUN-SL) was developed. In the third phase, psychometric properties of the CaSUN-SL were evaluated.

### Translation and adaptation of the CaSUN

A written permission from the Australian developers [12] to translate CaSUN was obtained. Next, two researchers working at the department independently translated the original version of the CaSUN into the Slovenian language. An expert team that consisted of an academic psychologist and a physician specializing in family medicine resolved any discrepancies in the translation. This resulted in a pre-final version of the CaSUN-SL. The translated version of CaSUN was translated back to English by two bilingual translators whose mother tongue is Slovenian with no medical background. The English version did not differ from the Slovenian version of the CaSUN. The pre-final version of the CaSUN-SL was proof-read.

The preliminary back-translated version of the CaSUN was pilot-tested on ten cancer survivors to ensure that the translated version was able to maintain its equivalence in a real situation. Selected patients primarily addressed scoring instructions and provided valuable feedback for the content and meaning of the adapted questionnaire. Each cancer survivor was asked if there was anything that was unclear. Feedback from cognitive interviewing served as a discussion point with the European Breast Cancer Coalition—Europa Donna Slovenia about the relevance of questions for a Slovenian cultural environment. The item 17 (“Due to my cancer, I need help accessing legal services”) was suggested to be excluded and additional text (“and employment-related legal services”) was added to the item 14 (“I need assistance with getting and/or maintaining employment…”). Additionally, some minor changes regarding comprehensibility and cultural relevance were made (e.g., the item 16 “Due to my cancer, I need help getting life and/or travel insurance” was changed to “Due to my cancer, I need help getting life insurance”).

### Participants and procedure

Cancer survivors were recruited from 30 family practices using a convenience sampling method. Each participating physician selected 15 adult cancer survivors’ post-treatment from medical records. The inclusion criteria included (1a) being diagnosed with cancer, (2a) completed surgery, radiotherapy, and/or chemotherapy, (3a) age of 18 or older and (4a) enough proficiency in the Slovenian language to complete the scale. Exclusion criteria included having (1b) a terminal illness and cancer recurrence, (2b) bedridden patient, (3b) current pregnancy, and (4b) diagnosis of neurological disease or a history of significant trauma.

Data were collected from June 2019 to October 2020. Participants were approached via a phone number provided by the physician. Those who agreed and signed the informed consent completed the self-administered questionnaire. Participants were fully informed about the purpose of the study and their right to refuse to participate at any stage. Data were collected from June 2019 to October 2020. One week after the first administration of the questionnaire, 45 clinically stable participants were asked to complete the CaSUN once again. Clinical stability of the participants was checked using the Hospital Anxiety and Depression Scale (HADS; scores higher than 10 in each subscale were excluded from the re-test analysis), following the COSMIN checklist (July 2019 version) [42].

### Instruments

Sociodemographic characteristics included age, gender, marital status, education, employment status and place of living. Clinical and behavioural characteristics included smoking status (smoker, non-smoker, smoking in the past), cancer type and stage, time since the end of the treatment, and type of treatment(s). See Table 1 for coding information.

**Table 1:** Sociodemographic, clinical and psychological characteristics of the sample

Variable	n	%
Age (M = 57.3; SD = 12.6, range = 28–87)		
Gender		
Male	62	21.0
Female	233	79.0
Marital status (n, %)		
Single	27	9.2
Married	167	57.2
Partnered	47	16.1
Divorced	16	5.5
Widowed	35	12.0
Educational status (n, %)		
Primary education	16	5.4
Lower/preparatory vocational education	69	3.4
General secondary education	84	28.5
Higher general secondary education	96	32.5
Post-graduate education	23	7.8
Employment status (n, %)		
Unemployed	14	4.8
Full-time employed	94	32.0
Half-time employed	49	16.7
Retired	122	41.5
Cancer type		
Breast cancer	148	51.0
Colon cancer	18	6.2
Lymphoma	19	6.6
Melanom	9	3.1
Others	93	32.1
Type of primary treatment		
Surgery (S)	65	22.3
S + Chemotherapy (CT)	45	15.5
S + Radiotherapy (RT)	45	15.5
S + CT + RT	102	35.1
Time since the end of primary treatment in years (M = 6.7; SD = 12.6; range = 0–33)		
Psychological distress		
Symptoms of anxiety (M = 6.09; SD = 3.8)		
Symptoms of depression (M = 6.23; SD = 3.6)		
Health-related quality of life		
EQ-5D-Index (M = 0.78; SD = 0.19)		

*EQ-5D* The EuroQol five-dimension questionnaire

*The Cancer Survivors’ Unmet Needs (CaSUN- SL) scale c*onsists of 34 items and measures cancer survivors related needs, six positive change items, and an open-ended question. Participants described whether the item was met, unmet or if the item was not applicable to them. If an item was unmet, they needed to describe the intensity of the unmet need, this was then scored as weak, moderate or strong [12]. The CaSUN-SL can be scored in terms of items or domains as a sum of unmet, met, total need, and/or strength of the need. The total score is obtained by summing all 34 items, where a higher score indicates greater needs. In our study we analysed the unmet, met and total CaSUN-SL score.

*The Hospital Anxiety and Depression Scale (HADS)* measures symptoms of anxiety and depression. It includes seven depression items and seven anxiety items on a 4-point scale. The scoring range is from 0 to 21 in each scale, where scores 0–7, 8–10, and 11–21 indicate non-case, borderline, and clinical case, respectively [43]. The Cronbach’s alpha of the Slovenian version of the HADS tested in a Slovenian sample of female cancer patients was 0.82 for depression, and 0.91 for anxiety [44]. In our study, we analysed the anxiety and depression subscales.

*The EuroQol Five-Dimension questionnaire (EQ-5D)* is designed to measure health-related quality of life [45]. It consists of two parts. In the first part, a participant describes their current health status, which is presented by a visual analogue scale (EQ-VAS) with 0 and 100 representing the worst and optimum health they can imagine. In the second part, a participant describes the (non) severity of the following activities: mobility, self-care, usual activities, pain/discomfort, and anxiety/depression. Severity can be rated as no problems, some problems, and extreme problems. The Cronbach’s alpha of the Slovenian version of the EQ-5D tested on a Slovenian sample of elderly Diabetes Mellitus Type 2 patients was 0.73 [46]. In this study, we included the EQ-5D-Index which was calculated according to the Slovenian population norm (data not published yet).

The 14-item Resilience Scale (RS-14) is a 14 items scale that measures the level of resilience. It uses a 7-point Likert Scale (1—strongly agree, 7—strongly disagree). The total score ranges from 14 to 98 points with higher score indicating higher resilience. Scoring guidelines proposed by Wagnild and Young [47] propose that the total score bellow 64 indicates low resilience, moderate wen the total score is 64–73, and high when the score is higher than 74. The Cronbach’s alpha for the Slovenian cancer survivors’ population was found to be excellent (α = 0.96). In this study the total RS-14 score was used.

### Data analysis

All statistical analyses except for model fit testing were performed using SPSS Software version 24 for Windows (SPSS, Chicago, IL, USA). For model fit testing, STATA Software version 14 (STATA Corp, College Station, TX) was used. Missing data were approached according to the CaSUN scoring manual [12]. The analysis was kept similar to the original study and the reports that followed [12, 20, 21, 23, 27].

The descriptive statistics method was used to describe the sample, using the mean, standard deviation and frequencies. The sample was split and both Exploratory Factor Analysis (EFA) and Confirmatory Factor Analysis (CFA) were performed, firstly to explore and secondly to confirm the identified factors. Factor structure was explored with Sample 1, whereas EFA (Maximum likehood using Oblimin with Kaiser Normalization rotation) was used to determine factor loadings. Items were kept if loaded with > 0.30. Sample 2 was used to confirm the established factors. Model fit was considered as acceptable if the values of the Tucker-Lewis Index (TLI) and Comparative Fit Index (CFI) were equal or greater than 0.90, and if the values of the Root Mean Square Error of Approximation (RMSEA) and Standardized Root Mean Residual (SRMR) were equal or lower than 0.09 [48].

The quality of the psychometric properties of the CaSUN-SL were measured with internal consistency, test–retest reliability and construct validity. Internal consistency was evaluated with the Cronbach α coefficient, and the test–retest reliability with the Interclass Correlation Coefficient (ICC; two-way mixed, absolute agreement) and t-Student test for dependent samples. Values on the ICC were evaluated as values of poor (0.5), moderate (0.5–0.75), good (0.75–0.90) and excellent reliability [49]. Construct validity was analysed with the Pearson’s correlation coefficient, comparing CaSUN-SL with the HADS subscales for anxiety and depression, and all EQ-5D subscales, including the total score. In addition, using univariate analysis correlations between CaSUN-SL and age, gender, marital status, type of cancer and time since treatment were evaluated. Strength of the association was interpreted using the Dancey and Reidy’s guidelines (0.1–0.3 weak, 0.4–0.6 moderate, 0.6–0.9 strong) [50, 51].

## Results

From 450 sent questionnaires, 295 were completed successfully and were analysed (65.6% response rate). Sociodemographic, clinical, behavioural, and psychological characteristics of the participants are shown in Table 1.

The majority of our sample were female (79.0%) with a mean (SD) age of 57.3 years. Over half of the participants were diagnosed with breast cancer (51.0%) and were at the time of the assessment on average 6.7 (SD = 12.6) years after the end of the treatment.

### Factor analyses

The total sample of 295 cancer survivors was randomly split into Sample 1 for EFA (n = 147) and Sample 2 for CFA (n = 148) to explore the factor analysis (EFA) and further to confirm it (CFA).

For the EFA, Items 13–17 and item 35 were not included in the analysis because they were endorsed by less than 10.0% of the participants or loading less than 0.30. However, they were all included in the final version of the questionnaire in the total needs score, as they were recognized as clinically and theoretically relevant. Results of the EFA (see Table 2) showed that one item (Item 27) in Existential Survivorship, one (Item 11) in Comprehensive Care, and one (Item 23) in Relationships fell into a different category than reported in the original study [12]. The category from the original CaSUN named “Quality of Life domain” is in the CaSUN-SL re-named as “Psychological and Emotional Support”, as it reflects the newly included factors much better. Item 10 (“Reduce stress”) loaded high on both domains, the Comprehensive Care domain (0.509) and the Psychological and Emotional Support domain (0.436), but was decided to be included in the latter as it fits better theoretically. The total variance explained in the study was 68.4%. Results on factor loadings compared to the original study and the final CaSUN-SL model are available in Table 2.

**Table 2:** Results of factor analysis of CaSUN-SL and its categorization

CaSUN	CaSUN-SL					
Scale	Item no. and description	Factor 1: ES	Factor 2: CC	Factor 3: PES	Factor 4: RE	Factor 5: IN
ES	32. Survivor expectations	**0.877**				
ES	31. Acknowledging the impact	**0.850**				
ES	25. Handle social/work situations	**0.649**				
ES	26. Changes to my body	**0.569**				
ES	34. Spiritual beliefs	**0.513**				
RE	27. Problems with sex life	**0.466**				
ES	24. Talk to others	**0.302**				
CC	6. Manage health with teams		**0.940**			
CC	8. Complaints addressed		**0.741**			
CC	7. Doctors talk to each other		**0.698**			
CC	5. Local health care services		**0.688**			
CC	4. Best medical care		**0.619**			
CC	9. Complimentary therapy		**0.588**			
QL	11. Manage side effects		**0.301**			
ES	29. Move on with my life			**0.803**		
ES	30. Changes to beliefs			**0.661**		
ES	19. Recurrence concerns			**0.616**		
ES	20. Emotional support			**0.567**		
ES	33. Decision about my life	0.310		**0.442**		
ES	10. Reduce stress		0.509	**0.436**		
QL	12. Changes to quality of life			**0.340**		
RE	22. Impact on my relationship				**0.720**	
RE	21. Support partner/family				**0.660**	
ES	23. New relationships			0.412	**0.354**	
IN	1. Up to date information					**0.681**
IN	3. Understandable information					**0.641**
IN	2. Information for others					**0.626**
	Eigen values	12.861	3.171	1.146	1.084	0.879
	% of variance	45.931	11.324	4.091	3.871	3.140
	Cumulative %	45.931	57.255	61.347	65.218	68.358

Bolded factor loadings in each category form a single factor

Maximum Likelihood Method*,* Oblimin with Kaiser Normalization in 7 iterations. With 5 factors and factor loading > .30

*ES* Existential Survivorship. *QL* Quality of Life. *CC* Comprehensive Cancer Care. *RE* Relations. *IN* Information. *PES* Psychological and Emotional Support

**Fig. 1:**
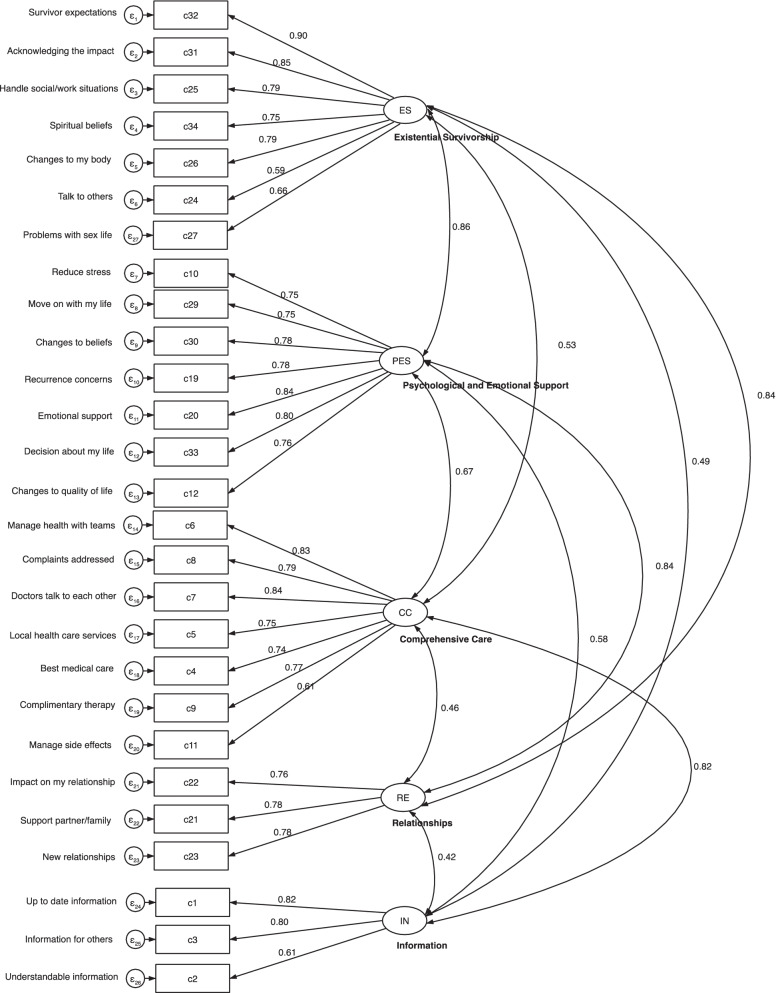
Standardized regression coefficients and correlations between errors of the CaSUN-SL

For the CFA, the goodness-of-fit of the obtained model was not firmly confirmed. The model (Chi square to df = 1.92, *p* < 0.001) showed values of CFI (0.890) and TLI (0.876) that are smaller than the recommended values of SRMR (0.075) and RMSEA (0.082) that meet the criteria for model fit. However, when applying less strict guidelines (i.e. TLI and CFI values close to 0.90 indicate a good model fit) [52], the criteria would be met. Nevertheless, the results should be taken with caution. Standardized path coefficients are shown in Fig. 1. A significant correlation among five CaSUN-SL domains exist, ranging from 0.42–0.86.

### Internal consistency and test–retest reliability

Internal consistency of the CaSUN-SL total score showed an excellent reliability with a Cronbach’s α of 0.94. Internal consistency of the five domains is shown in Table 3, ranging from 0.71–0.88.

**Table 3:** Internal consistency of the CaSUN-SL scale and its subscales

Scale/subscale	Cronbach's alpha value
CaSUN-SL (34 items)	0.940
Existential survivorship	0.862
Comprehensive Care	0.821
Psychological and emotional support	0.877
Relationship	0.748
Information	0.705

*CaSUN-SL* The Slovenian version of the Cancer Survivors’ Unmet Needs

Test–retest reliability was performed in a group of 31 (retention rate at the re-test assessment: 68.8%) participants with an average of 7–10 days after the first administration of the questionnaires. Correlations between time 1 and time 2 (Pearson's correlation test) between every item (34 items) showed moderate-high stability over time (r = 0.54–0.97; *p* < 0.01). ICC values ranged from 0.71 (*p* < 0.001) to 0.98 (*p* < 0.001). Test–retest reliability for the 34 items showed good to excellent values, for met (r = 0.651, *p* < 0.001; ICC = 0.79, *p* < 0.001), unmet (r = 0.91, *p* < 0.001; ICC = 0.95, *p* < 0.001), total (r = 0.91, *p* < 0.001; ICC = 0.95, *p* < 0.001), and strength of the need (r = 0.88, *p* < 0.001; ICC = 0.93, *p* < 0.001).

Construct validity.

Table 4 shows the correlations between met, unmet and total needs of the CaSUN-SL and selected variables. Construct validity showed that age was significantly correlated with met (r = 0.13, *p* < 0.05), unmet (r = 0.29, *p* < 0.001) and total needs (0.37, *p* < 0.001), indicating that younger patients have more met and unmet needs. Significant correlations were found between unmet needs and gender (r = 0.14, *p* < 0.05), indicating that female patients have more unmet needs; type of cancer (r =  − 0.19, *p* < 0.001), indicating that breast and lymphoma cancer survivors have the highest unmet needs; time since treatment (r =  − 0.20, *p* < 0.001), indicating that with the less time that has passed since the end of the primary treatment, the more unmet needs are observed; and stage of cancer (r = 0.20, *p* < 0.001), indicating that patients who were diagnosed with more advanced cancer reported more unmet needs.

**Table 4:** Construct validity between CaSUN-SL and selected variables

Variable	Met needs	Unmet needs	Total needs
Age	− 0.13*	− 0.29**	− 0.37**
Gender	0.10	0.14*	− 0.04
Marital status	0.03	− 0.91	− 0.06
Employment	0.03	− 0.26**	− 0.22**
Type of cancer	0.07	− 0.19**	− 0.13*
Stage of cancer	0.08	0.20**	0.25**
Time since treatment	− 0.13	− 0.20**	− 0.27**
Anxiety (HADS-A)	0.07	0.49**	0.49**
Depression (HADS-D)	0.05	0.44**	0.43**
Quality of life (EQ-5D-Index)	0.07	0.27**	0.30**
Resilience (RS-14)	0.02	− 0.47**	− 0.41**

The CaSUN-SL included 34 items that were retained after the cultural adaptation of the translated Slovenian version

^*^p ≤ 0.05; **p ≤ 0.001

The subscales HADS anxiety and HADS depression, and the EQ-5D-total were all positively correlated with the unmet and total needs (see Table 4), indicating that cancer patients with higher unmet and total needs have more symptoms of anxiety, depression, and more problems on the quality-related components (i.e., mobility, self-care, usual activities, pain/discomfort, and anxiety/depression). On the contrary, EQ-5D subscale EQ-VAS and RS-14 were found to be negatively correlated to the unmet and total needs (Table 4), indicating that cancer patients with higher unmet and total needs experience lower self-perceived health and resilience.

## Discussion

This study reports on the translation and validation of the Slovenian version of the CaSUN. Overall, our results indicate that the CaSUN-SL has satisfactory psychometric properties in a Slovenian sample of cancer survivors.

The majority of items included in the original factors were in accordance with the original study [12], although the concurrence was not absolute. The authors' original domain Quality of life domain was in the CaSUN-SL re-named as the Psychological and emotional domain. As argued in previous reports [20, 53], items of Existential survivorship and Quality of life domain may be overly associated, as the existential well-being of a cancer survivor is crucial for their quality of life. Thus, the EFA in our sample has revealed that it would be better to name Factor 3 Psychological and emotional domain instead of Quality of life. The new re-named domain pertains to the psychological and emotional support that can be offered to the cancer survivor (e.g., how to move on with their life, reducing stress, addressing recurrence concerns), whereas the domain Existential survivorship pertains more to the patient’s life perspective as a cancer survivor (e.g., survivor expectations, acknowledging the impact, handle social/work situations). Nevertheless, there still exists a high correlation between those two domains (r = 0.86; see Fig. 1), thus the relationship warrants further investigation. The other three domains (Comprehensive care, Relationship and Information) remained intact in most parts. The goodness-of-fit index altogether suggests a reasonable and yet suboptimal model-data fit; these should be interpreted with caution as guidelines across the literature are not uniform. Model fit indexes were reported in two previous validation studies of the CaSUN, where in both the model fit was found to be acceptable [27, 39]. It should be noted that their samples for CFA were much higher than in our study (n for CFA > 300).

The internal consistency reported in our study was found to be fair to excellent for specific domains and comparable with other studies [12, 20, 27]. The test–retest reliability showed moderate to high stability over time and is in accordance with previous studies [20, 27, 39] that had overcome the limitations of the original study [12]. The rationale for choosing a shorter time between the first and second assessment (7–10 days) was a report from the systematic literature review stating that unmet needs decrease over time [7]. Test–retest reliability is defined as the consistency of scores obtained by the same person, same test and at a different time with the goal of differentiating true score variances from random measurements errors [54]. It should be measured on a person with a minimal risk of experiencing a sudden clinical worsening or a significant event. To reduce the probability of change, we included only participants that were clinically stable (as confirmed with a HADS score lower than 10) and minimizing the time length between the measurements. Our pack of questionnaires included several other questionnaires, thus lowering the risk of remembering previous answers.

Results on the construct validity showed significant correlations in expected directions. In line with previous studies [7, 8], cancer survivors with more symptoms of anxiety, depression and more quality-related problems reported more unmet needs. Patients with higher resilience and better self-perceived health reported fewer unmet needs. Regarding demographic characteristics, younger and female patients reported more unmet needs. Younger cancer survivors have higher expectations about their physical health and needs that pertain to fertility [55–57]. It is very important to acknowledge that younger cancer survivors have unique needs that need to be recognized in the healthcare system. Not only during the treatment but—even more importantly—after the safety net of the treatment ends. Further, the literature reporting gender differences is scarce, although the majority of studies have been conducted with females due to a higher response rate. Our results with an albeit limited number of males indicate that females have more unmet needs. Regarding cancer-related characteristics, breast and lymphoma cancer survivors, survivors with more advanced stages of the disease, and those with less time passing since the end of the treatment, reported more unmet needs. These findings are in line with previous reports [7, 8, 58].

Future work should be oriented towards assessing the prevalence and factors associated with a higher level of unmet needs. Our study provides a first step in identifying variables that should be included in the multivariate regression models in the future studies (e.g., age, gender anxiety, depression, quality of life). Further, cancer survivors identified with a higher level of unmet needs should be offered targeted interventions to decrease their levels of unmet needs. In the literature, fear of cancer recurrence and a desire of ‘being informed about the things you can do to help yourself get well’ are two most commonly endorsed unmet needs [7]. Psychological interventions for addressing fear of cancer recurrence have emerged in the past years and show strong effect in the latest meta-analysis [59]. Interventions addressing the unmet need for information are usually merged into ‘self-management interventions’ and besides medical management, also include the role and emotional management, which have as well been reported as critical unmet needs [60]. Due to the variety of intervention designs, psychoeducational and theoretical contents, effect is not possible to calculate. This hinders translation of such intervention to the clinical practice [61].

The study’s strength is using standardized guidelines for translating the scale and considering cultural adaption. The results are based on the robust analyses, performing EFA, CFA, internal consistency, test–retest reliability, and construct validity. Although this study is presented with a smaller sample size (N = 295) than those observed in previous studies, the results indicate a reasonably reliable conclusion. Nearly 80% of the participants were female, thus a potential bias may exist due to an over-representation of females and the small subsample of male cancer survivors. Additional bias regarding socio-economic and ethnic diversity was planned to be minimised with the recruitment of patients from urban, suburban, and rural areas. Due to the General Data Protection Regulation (EU), it was not possible to conduct the responders and non-responders’ analysis.

## Conclusions

Due to a growing population of cancer survivors, it is essential to explore and identify their needs. Valid instruments for assessing cancer survivors' needs are the first step in addressing their unmet needs. Slovenian version of the CaSUN with 34 items and five factors represents the first translated and validated measure in Slovenia and the first step in addressing unmet needs. The CaSUN-SL can now be used to assess the prevalence of, and correlates with, unmet needs in a variety of cancer survivors’ populations in Slovenia. This will enable healthcare professionals and policy makers to focus on tailored interventions that will target the most critical unmet needs.

## Data Availability

The datasets used and/or analysed during the current study are available from the corresponding author on reasonable request.

## Abbreviations

CaSUN: The Cancer Survivors’ Unmet Needs
SUNS: The Survivor Unmet Needs Survey
CNQ-YP: The Cancer Needs Questionnaire-Young People
CCSS-NAQ: The Needs Assessment Questionnaire
SCNS-34: The 34-item Supportive Care Needs Survey
HADS: The Hospital Anxiety and Depression Scale
RS-14: The 14-item Resilience Scale
EQ-5D: The EuroQol five-dimension questionnaire
EQ-VAS: The EuroQol Visual Analogue Scale
EFA: Exploratory Factor Analysis
CFA: Confirmatory Factor Analysis
TLI: Tucker-Lewis Index
CFI: Comparative Fit Index
RMSEA: Root Mean Square Error of Approximation
SRMR: Standardized Root Mean Residual
ICC: Interclass Correlation Coefficient
